# Supplementary figures and images for: Effects of oxytocin administration and conditioned oxytocin on brain activity: An fMRI study
Source: PLoS One. 2020 Mar 19;15(3):e0229692. doi: 10.1371/journal.pone.0229692 (PMC7082015; doi:10.1371/journal.pone.0229692)

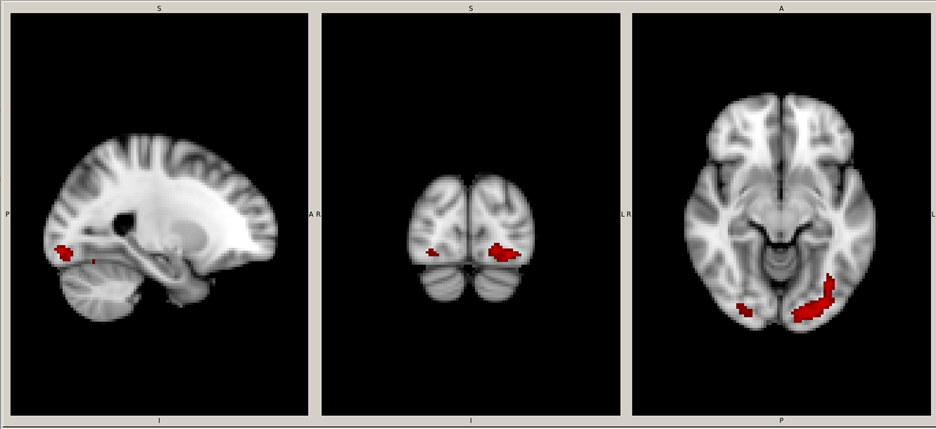

Supplement: S1 Fig — (TIF) [file pone.0229692.s001.tif]

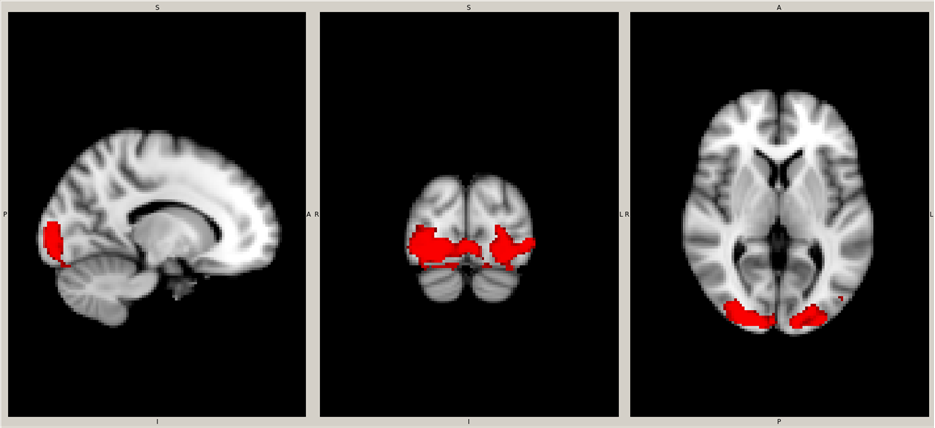

Supplement: S2 Fig — (TIF) [file pone.0229692.s002.tif]

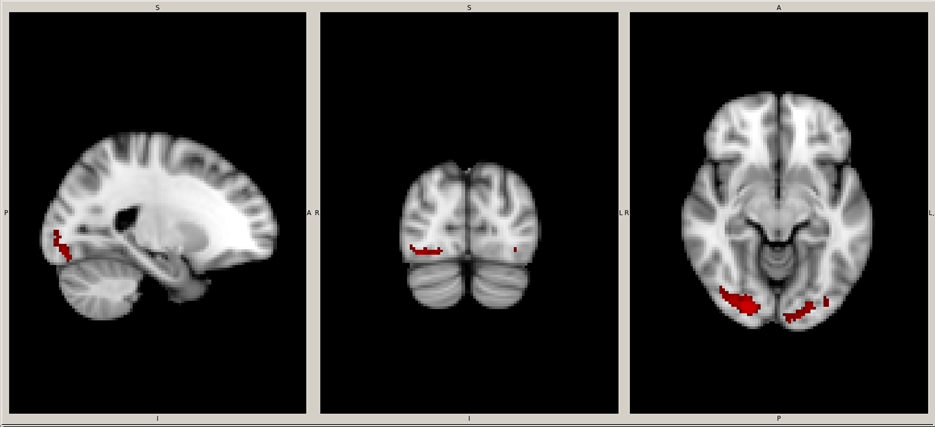

Supplement: S3 Fig — (TIF) [file pone.0229692.s003.tif]

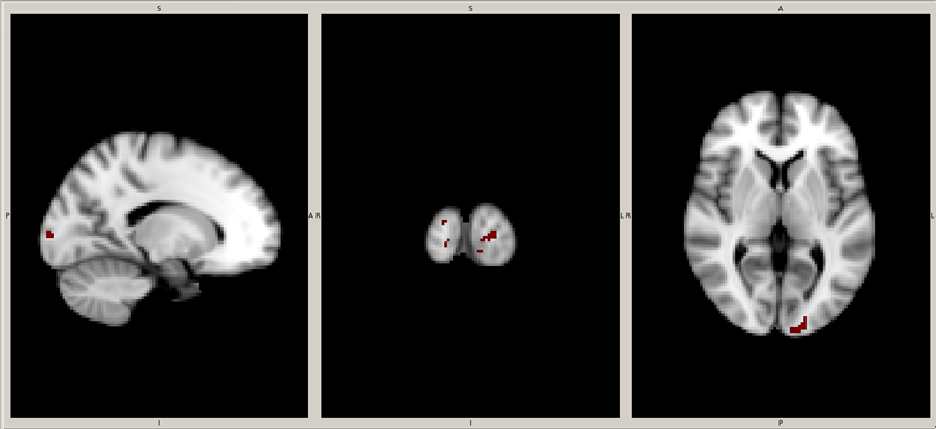

Supplement: S4 Fig — (TIF) [file pone.0229692.s004.tif]

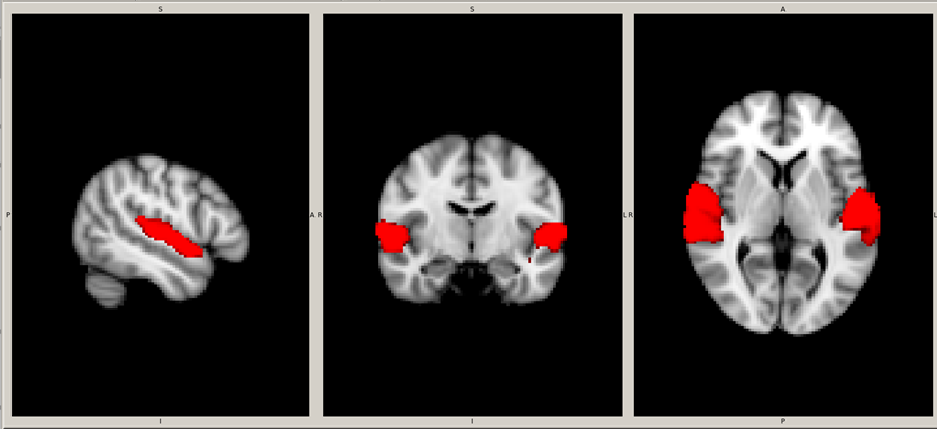

Supplement: S5 Fig — (TIF) [file pone.0229692.s005.tif]

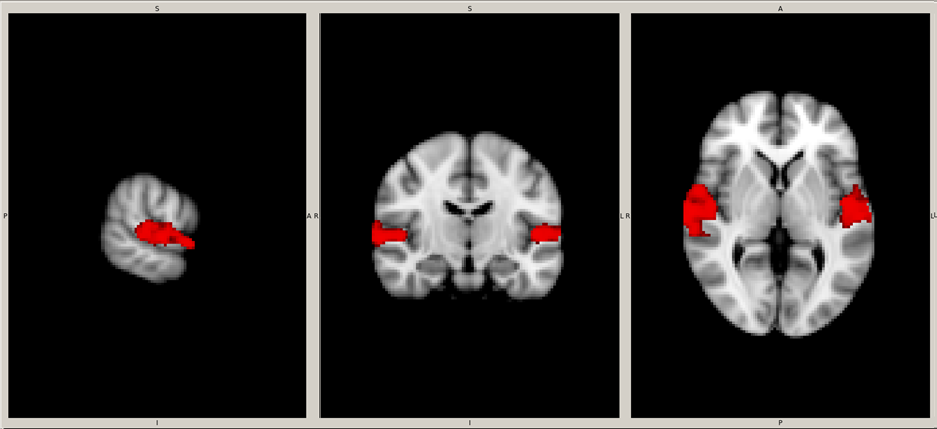

Supplement: S6 Fig — (TIF) [file pone.0229692.s006.tif]

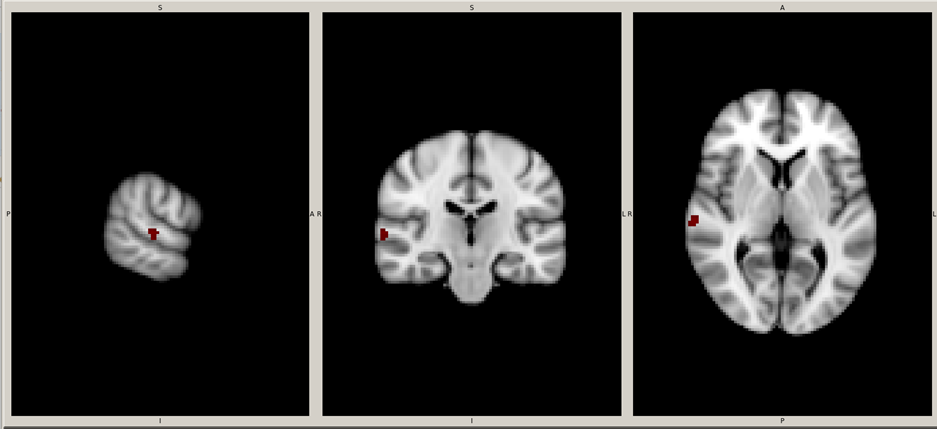

Supplement: S7 Fig — (TIF) [file pone.0229692.s007.tif]

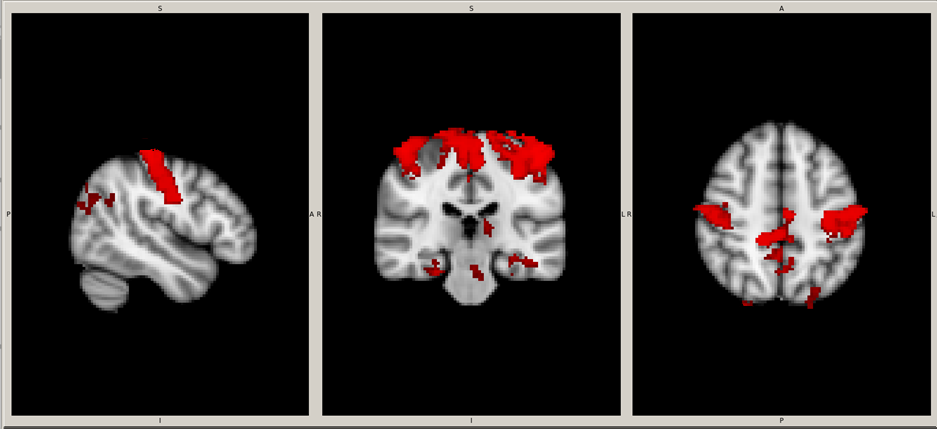

Supplement: S8 Fig — (TIF) [file pone.0229692.s008.tif]

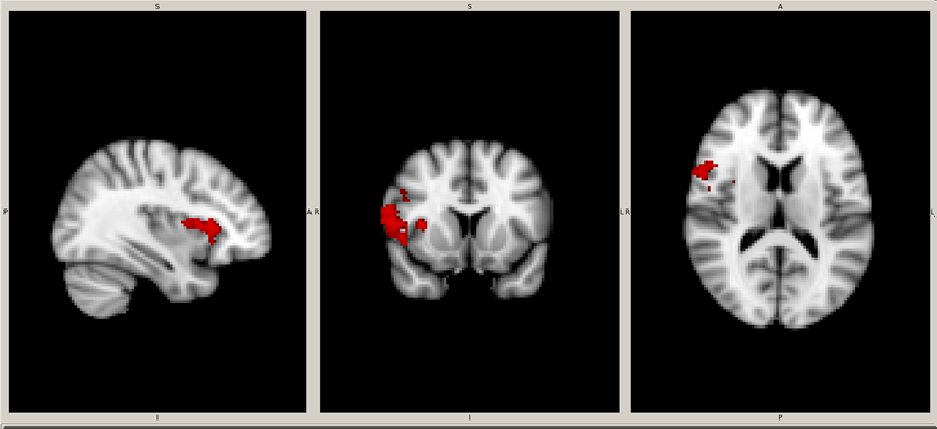

Supplement: S9 Fig — (TIF) [file pone.0229692.s009.tif]

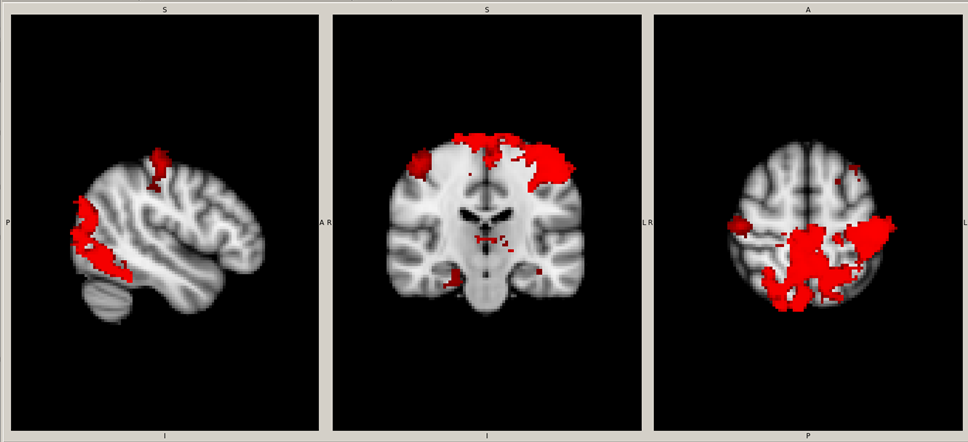

Supplement: S10 Fig — (TIF) [file pone.0229692.s010.tif]

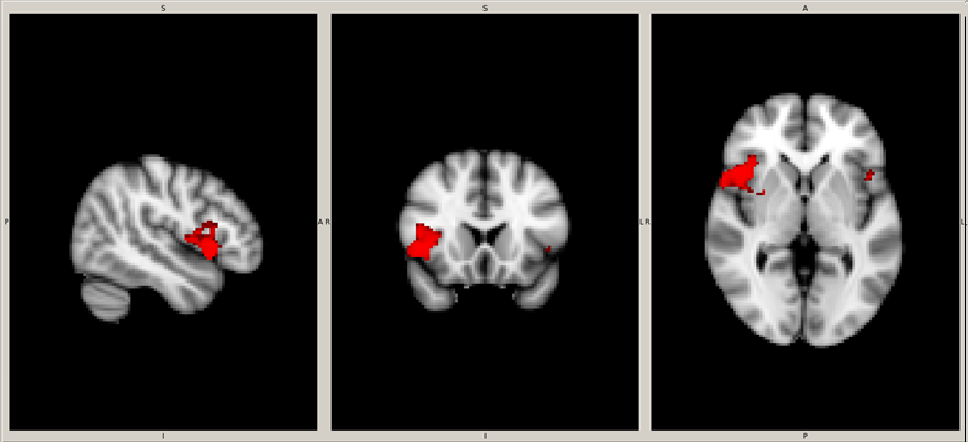

Supplement: S11 Fig — (TIF) [file pone.0229692.s011.tif]

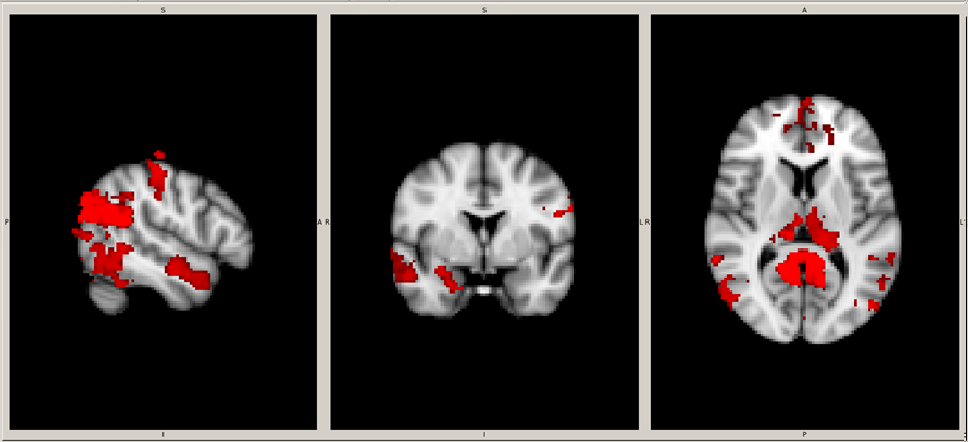

Supplement: S12 Fig — (TIF) [file pone.0229692.s012.tif]

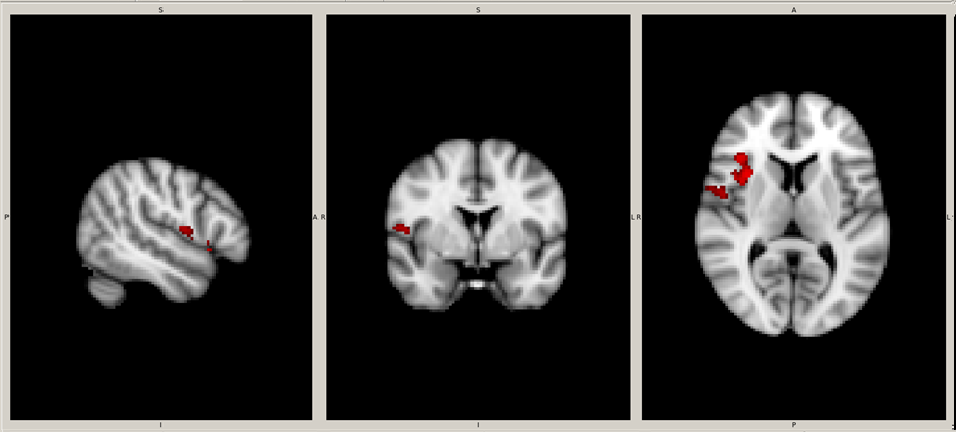

Supplement: S13 Fig — (TIF) [file pone.0229692.s013.tif]
